# Clinical Factors Associated with a Shorter or Longer Course of Antibiotic Treatment in Patients with Exacerbations of Bronchiectasis: A Prospective Cohort Study

**DOI:** 10.3390/jcm8111950

**Published:** 2019-11-12

**Authors:** Giulia Scioscia, Rosanel Amaro, Victoria Alcaraz-Serrano, Albert Gabarrús, Patricia Oscanoa, Laia Fernandez, Rosario Menendez, Raul Mendez, Maria Pia Foschino Barbaro, Antoni Torres

**Affiliations:** 1 Medical and Surgical Sciences Department, Institute of Respiratory Disease, University of Foggia, 71121 Foggia, Italy; giuliascioscia84@gmail.com (G.S.); mariapia.foschino@unifg.it (M.P.F.B.)); 2 Institute of Respiratory Disease, Hospital Clínic of Barcelona, 08036 Barcelona, Spain; RAMARO@clinic.cat (R.A.); OSCANOA@clinic.cat (P.O.); 3 Fundació Clínic per la Recerca Biomèdica (FCRB), Institut d’Investigacions Biomèdiques August Pi i Sunyer (IDIBAPS), Biomedical Research Networking Center on Respiratory Diseases (CIBERES), Hospital Clínic of Barcelona, 08036 Barcelona, Spain; victoriaalcarazserrano@gmail.com (V.A.-S.); GABARRUS@clinic.cat (A.G.); LFERNAN1@clinic.cat (L.F.); 4 Pneumology Department, La Fe University and Polytechnic Hospital, La Fe Health Research Institute, 46026 Valencia, Spain; rosmenend@gmail.com (R.M.); rmendezalcoy@gmail.com (R.M.)

**Keywords:** antibiotic course, bronchiectasis, exacerbations, severity

## Abstract

Background: Bronchiectasis exacerbations are often treated with prolonged antibiotic use, even though there is limited evidence for this approach. We therefore aimed to investigate the baseline clinical and microbiological findings associated with long courses of antibiotic treatment in exacerbated bronchiectasis patients. Methods: This was a bi-centric prospective observational study of bronchiectasis exacerbated adults. We compared groups receiving short (≤14 days) and long (15–21 days) courses of antibiotic treatment. Results: We enrolled 191 patients (mean age 72 (63, 79) years; 108 (56.5%) females), of whom 132 (69%) and 59 (31%) received short and long courses of antibiotics, respectively. Multivariable logistic regression of the baseline variables showed that long-term oxygen therapy (LTOT), moderate–severe exacerbations, and microbiological isolation of *Pseudomonas aeruginosa* were associated with long courses of antibiotic therapy. When we excluded patients with a diagnosis of community-acquired pneumonia (*n* = 49), in the model we found that an etiology of *P. aeruginosa* remained as factor associated with longer antibiotic treatment, with a moderate and a severe FACED score and the presence of arrhythmia as comorbidity at baseline. Conclusions: Decisions about the duration of antibiotic therapy should be guided by clinical and microbiological assessments of patients with infective exacerbations.

## 1. Background

Bronchiectasis is a structural respiratory disease characterized by permanently dilated bronchi. The pathogenesis consists of chronic bronchial infection, neutrophil-mediated inflammation, structural lung disease, and impaired mucociliary clearance. This pathology relapses and remits during the course of the disease, leading to increased bacterial load and recurrent exacerbations [1]. As the frequency of exacerbations increases, there is an associated reduction in the forced expiratory volume in 1 s, an increased severity of lung disease on computed tomography (CT), and an increase in chronic infection with *Pseudomonas aeruginosa* and *Haemophilus influenza* [2]. In addition, more severe and frequent exacerbations are associated with a worse quality of life, more hospital admissions, higher mortality, and increased economic burdens [3].

In a recent consensus, experienced clinicians proposed a working definition of an exacerbation of bronchiectasis for use in clinical research [4]. Most therapeutic interventions focus on reducing exacerbations to improve short- and long-term outcomes, so it is recommended practice to initiate oral or intravenous antibiotic treatment based on knowledge of the likely causative organism. Existing guidelines predominantly recommend empirical antibiotic choices based on whether *P. aeruginosa* is being targeted [5].

To date, the optimal duration of antibiotic treatment for exacerbations of bronchiectasis has not been systematically studied. The practice of 14 days’ therapy for infective exacerbations was extrapolated from the treatment approach used for cystic fibrosis (CF), without any definitive evidence for the approach in non-CF bronchiectasis populations. In the guidelines on non-CF bronchiectasis by the European Respiratory Society [6], the authors systematically reviewed the literature comparing short (<14 days) and long (14–21 days) treatment durations. They reported only one small study in which the outcomes were similar at 7 and 14 days [7].

We hypothesized that clinical and microbiological characteristics exist that can be associated with the optimal duration of antibiotic treatment during an exacerbation. In this study, we specifically aimed to identify the factors associated with long-course antibiotic treatment for exacerbations of bronchiectasis.

## 2. Materials and Methods

### 2.1. Study Design

A prospective observational study of consecutive adult patients (>18 years) with bronchiectasis exacerbations was conducted among patients treated by the pulmonology services of two tertiary care university hospitals in Spain between 2011 and 2015. The local ethics committees approved the study (Hospital Universitario y Politécnico La Fe, Valencia N° 2011/0342; Hospital Clínic, Barcelona N°2013/8071), which was carried out according to the principles of the Declaration of Helsinki. Informed consent was considered unnecessary due to the observational design. There were no specific requirements for data collection, with all data resulting from routine clinical activity and from tests and procedures ordered by attending physicians who were not involved in the study. All patient data were anonymized.

### 2.2. Patients

The main inclusion criterion was a clinical history compatible with exacerbation, with diagnosis of bronchiectasis confirmed by high-resolution CT before recruitment. Disease etiology was established according to Spanish guidelines [8]. The following exclusion criteria were applied: (a) diagnosis of CF, ciliary dyskinesia, pulmonary interstitial disease, active tuberculosis, or non-tuberculosis mycobacterial infection during treatment; (b) exacerbation of any comorbidity; and (c) participation in any clinical trial that included changes in pharmacological treatment in the preceding 6 months.

### 2.3. Definitions

Exacerbations were defined according to the international consensus statement [4]. Only the first exacerbation diagnosed and treated with antibiotics during the study period was considered. Mild exacerbations were those treated with oral antibiotics as outpatients, whereas moderate to severe exacerbations were those requiring hospitalization, intravenous therapy, intensive care admission, and/or mechanical ventilation [9].

For comparison, two groups were formed according to the treatment duration chosen according to Spanish guidelines [8]: a short-course group (≤14 days) and a long-course group (15–21 days). We did not follow a standardized algorithm for the treatment decision-making. The two groups were formed according to the treatment duration chosen after an independent clinical decision made by the attending physician of the emergency department who were not involved in the study and determined the different therapeutic options and the course lengths at the onset depending on the etiologic microbiological diagnosis and severity of the exacerbation. Treatment was stopped once clinical success was achieved, which was the complete resolution or a reduction of signs and symptoms associated with the exacerbation without new signs and symptoms developing at the end of the treatment. Poor clinical response was defined as the persistence of ≥2 symptoms for >5 days at the end of the treatment [10].

### 2.4. Data Collection and Evaluation

We collected baseline clinical and demographic data, as well as bronchiectasis severity scores [3,11].

Current exacerbations that presented with a new infiltrate on chest X-ray were included. The decision to admit to hospital was made by the attending physician in the emergency department based on the presence or absence of acute findings consistent with a moderate to severe exacerbation. Blood and microbiological extractions were performed within 24 h of initial assessment.

### 2.5. Follow-Up

All surviving patients were re-examined 30 days and 1 year after hospital discharge or at the end of treatment in an outpatient clinic to assess their clinical response.

### 2.6. Statistical Analysis

Categorical variables were reported as numbers and percentages, whereas continuous variables were reported as medians and first to third quartiles. Group comparisons for categorical variables were conducted with the chi-square test or Fisher exact test, and comparisons for continuous variables were conducted using the non-parametric Mann–Whitney test. Unless stated otherwise, the significance level was set at 0.05 (two-tailed). All analyses were performed using IBM SPSS version 25.0 (IBM Corp., Armonk, USA).

Logistic regression analyses [12] were performed to examine the risks factors for long-course antibiotic treatment. We only included the following baseline variables when studying potential in the univariate analyses: gender; smoking habit; primary bronchiectasis etiology (e.g., asthma, chronic obstructive pulmonary disease (COPD), idiopathic, or post-infectious); use of long-term oxygen therapy (LTOT); use of oral corticosteroids; severity scores (e.g., the FACED score or Bronchiectasis Severity Index (BSI)), presence of a comorbidity (e.g., COPD, asthma, or arrhythmia); place of initial treatment during an acute episode; severity of acute exacerbation; presence of increased sputum purulence or fever; confirmed diagnosis of community-acquired pneumonia; and the microbiological isolation of *P. aeruginosa*, Methicillin-resistant *Staphylococcus aureus,* or polymicrobial etiology. Each risk factor was first tested individually before we added all risk factors that showed a univariate association (*p* < 0.10) to the multivariable model. Next, backward stepwise selection (*p*_in_ < 0.05, *p*_out_ > 0.10) was used to determine the factors associated with long antibiotic treatment courses. Odds ratios (ORs) and their 95% confidence intervals (CIs) were calculated.

A multiple imputation method [13] was used to handle missing data in the multivariable analyses. Multicollinearity was checked by calculating the variance inflation factor. The Hosmer–Lemeshow goodness-of-fit test was used to evaluate the adequacy of the model. In addition, the area under the receiver operating characteristic curve (AUC) was calculated for the ability of the final model to predict patients requiring long-course antibiotic treatment. Finally, to measure possible overfitting and instability of the selection variables in the final model, we performed internal validation using ordinary non-parametric bootstrapping with 1000 bootstrap samples and bias-corrected accelerated 95% Cis [14].

## 3. Results

### 3.1. Participants

**Figure 1:**
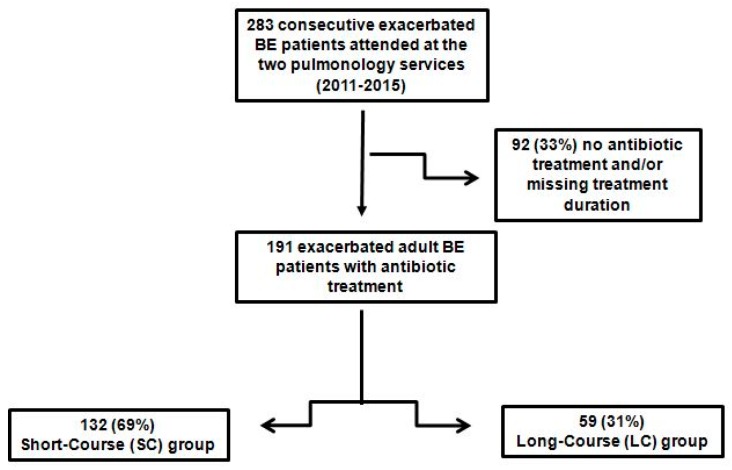
Chart for study enrollment.

Of the 283 exacerbated patients attended by the participating hospitals with exacerbations of bronchiectasis during the study period, 92 (33%) were excluded from the analysis because they did not receive antibiotic treatment, or the duration of the antibiotic treatment was not recorded. The population therefore comprised 191 patients with bronchiectasis exacerbations, of which most were women (57%; *n* = 108) and elderly (median age, 72 (63, 79) years). Participants were divided in the short-course group (69%; *n* = 132) and the long-course group (31%; *n* = 59) based on their antibiotic treatment duration, as decided by the attending physician (Figure 1).

### 3.2. Comparison of Patients by Treatment Duration Group

Table 1 shows a comparison of the baseline characteristics of patients in the short- and long-course groups. Patients with longer durations of antibiotic therapy received LTOT, had cylindrical or cystic bronchiectasis on high-resolution CT, had a higher incidence of chronic colonization with *P. aeruginosa*, had more exacerbations and hospitalizations due to bronchiectasis in the last year, and more often had a severe FACED score. Patients with short-term therapy more frequently had a mild FACED score. Twenty-six patients (13.6%) received LTOT at baseline. All of them had a severe BSI stage, 15 (57.7%) had a severe FACED score, while 4 (15.4%) presented a mild FACED score and 7 (26.9%) a moderate one (*p* < 0.001).

Table 2 shows data for patient characteristics during exacerbations between the treatment duration groups. Mild exacerbations were more frequent in patients receiving short-course therapy, whereas moderate to severe exacerbations were associated with longer courses of antibiotic therapy. The mean exacerbation and hospitalization lengths were also significantly higher in patients with longer courses of antibiotic treatment.

Overall, an etiologic microbiological diagnosis was obtained at baseline in 141 patients (74%). Patients with longer courses of antibiotic treatment more frequently had *P. aeruginosa* isolated and initially received three or more antibiotics compared with patients who received short-term therapy. We detected a change in the initial decision in 60 (31.4%) patients that corresponded to the cases in which the treatment had been started empirically according to previous cultures and then improved based on the current antibiogram.

### 3.3. Outcomes

Patients in the long-course group more often had poor clinical response to treatment (*p* = 0.003). However, no statistically significant differences were found between the two groups in terms of the history of exacerbations or hospitalizations during the 1-year follow-up. Similarly, the mortality rates during the exacerbation and at 30 days and 1 year of follow-up did not differ between the two groups (Table 3).

### 3.4. Factors Associated with Longer Courses of Antibiotic Treatment

**Figure 2:**
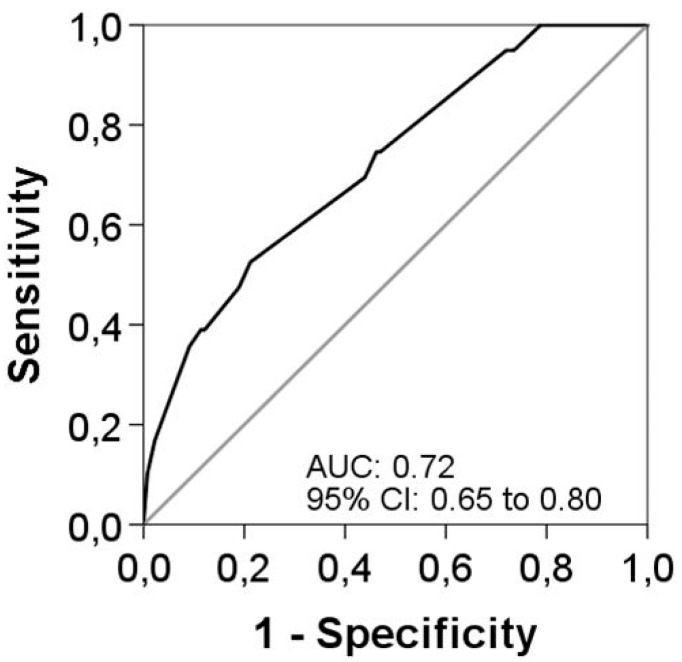
Area under the receiver-operator characteristic curve for the multivariable model.

Univariate logistic regression analyses were performed for the main characteristics of patients at baseline and during an acute exacerbation. Several variables were significantly associated with longer courses of antibiotic treatment (Table 4). However, when all significant variables were put into the multivariable model, only three factors remained as independent associated with longer antibiotic treatment. These were the use of LTOT (OR 2.51 (95% CI 1.02 to 6.22)), the presence of a moderate to severe exacerbation (OR 3.31 (95% CI 1.26 to 8.65)), and an etiology of *P. aeruginosa* confirmed by microbiological isolation (OR 2.89 (95% CI 1.43 to 5.82)). The AUC was 0.73 (95% CI 0.65 to 0.80) for this final model predicting longer antibiotic treatment duration (Figure 2). Internal validation by bootstrapping with 1000 samples demonstrated robust results. All three variables remained significant after bootstrapping, with small 95% CIs around the original coefficients.

### 3.5. Supplementary Analysis

We repeated all regression analyses after excluding patients with a diagnosis of community-acquired pneumonia (*n* = 49) and we have included the results in the Supplementary Material (Table S1). In the multivariable model, we found that an etiology of *P. aeruginosa* confirmed by microbiological isolation (OR 3.07 (95% CI 1.31 to 7.18)) remained as independent factor associated with longer antibiotic treatment, with a moderate FACED score (OR 2.97 (95% CI 2.09 to 8.14)), a severe FACED score (OR 4.31 (95% CI 1.49 to 12.42)), and the presence of arrhythmia as comorbidity at baseline (OR 0.19 (95% CI 0.05 to 0.73)). The AUC was 0.77 (95% CI 0.68 to 0.85) for this final model predicting longer antibiotic treatment duration (Figure S1). The outcomes were equal to those reported for the full cohort (Table S2).

## 4. Discussion

To the best of our knowledge, this is the first analysis of baseline patient, clinical, and microbiological characteristic associated with longer courses of antibiotic therapy for exacerbations of bronchiectasis. The main findings of the study are as follows. First, we showed that longer courses of antibiotic treatment were provided to patients treated with LTOT and who had a more severe disease at baseline. Second, we showed that moderate to severe bronchiectasis exacerbations lasting more days, and for which *P. aeruginosa* is isolated also tend to receive longer antibiotic courses. Third, we also showed that LTOT, moderate to severe exacerbations, and microbiological isolation of *P. aeruginosa* were associated with the need for longer courses of antibiotic treatment. These three aspects could help us to detect those patients with an exacerbation who require a longer course of antibiotic treatment.

The optimal duration of either oral or intravenous antibiotic therapy has not been studied for non-CF bronchiectasis, with current practice extrapolated from that used for CF. In the absence of any direct data comparing long and short courses, 14 days of treatment continues to be recommended in guidelines [2,15,16]. Some authors [17,18] have demonstrated a favorable impact with 14 days of intravenous antibiotic therapy for exacerbations, with improvements shown in sputum bacterial volume, markers of inflammation, the incremental walk test, and the St. George’s Respiratory Questionnaire. However, spirometry results remained unchanged. Collaco et al. [19] also observed that patients with CF who received longer courses of intravenous antibiotics tended to have worse lung function and they demonstrated that the greatest improvement in lung function may occur within the first week of therapy, indicating that courses lasting 14–21 days may provide no additional benefit.

Consistent with the recent guideline of the British Thoracic Society [20], we observed that patients treated with longer courses of antibiotics presented with more markers of severe disease than patients treated with shorter-term therapy. When we assessed the two main severity scores, the FACED score was milder in the short-course group and more severe in the long-course group. In the supplementary analysis, we showed that a moderate and a severe FACED score were independent factors associated with longer antibiotic treatment for patients with a bronchiectasis exacerbation without a diagnosis of community-acquired pneumonia. By contrast, no differences were found for the BSI score. FACED is an easy-to-use system that incorporates five dichotomized parameters to classify severity according to its 5-year prognosis [11]. Rosales et al. [21] demonstrated that severity stratified by FACED and BSI scores can show relevant differences in patient distribution. 

Concerning the microbiological results, we showed that prior chronic colonization with *P. aeruginosa* or etiologic isolation during a bronchiectasis exacerbation influenced the need for prolonged antibiotic treatment. Recently, Crisafulli et al. [22] also demonstrated that *P. aeruginosa* affected the length of hospitalization during exacerbations of COPD. We could explain the longer exacerbation and hospitalization durations of our patients in the long-course group by *P. aeruginosa* isolation in 31.4% of cases. It was also possible to explain the initial use of three or more antibiotics in 15 patients from the long-course group by the isolation of multidrug resistant *P. aeruginosa* in 15.2%.

Univariate analysis of the main characteristics at baseline and during exacerbations revealed several variables that were significantly associated with longer courses of antibiotic treatment. However, the multivariate analysis only confirmed that LTOT, the presence of a moderate to severe exacerbation, and the microbiological isolation of *P. aeruginosa* during an exacerbation were significant associated with longer treatment durations. In recent study, Sundh et al. [23] concluded that LTOT prescribed for 24 h each day did not decrease hospital admissions compared therapy for just 15–16 hin patients with oxygen-dependent COPD. Together with our finding, this could indicate that LTOT usage is associated with considerable healthcare costs. On the other hand, we can say that LTOT is a clinical factor that reflect disease severity. Previous studies have shown that *P. aeruginosa* isolation in bronchiectasis patients was associated with accelerated loss of lung function and increased daily sputum production [24], as well as a poorer quality of life, regardless of whether it is a cause or consequence of functional deterioration [25]. The findings of our study also suggest that longer antibiotic treatment during exacerbations is appropriate for patients with *P. aeruginosa* infection (with and without diagnosis of community-acquired pneumonia), consistent with the latest recommendations of the British Thoracic Society [20].

Finally, when we repeated all regression analyses after excluding patients with a diagnosis of community-acquired pneumonia, we found that the presence of arrhythmia as comorbidity at baseline appear a factor negatively associated with long-term therapy. A potential explanation is that attending physicians tended to shorten the course of antibiotic therapies to avoid cardiac complications in patients with a baseline arrhythmic comorbidity, or it could simply be a random response without any clinical significance. Future studies addressing the impact of vascular disease on bronchiectasis exacerbations are desirable.

The strengths of our study are the prospective inclusion of consecutive patients in two centers, the inclusion of clinically relevant variables, and the long-term follow-up. The study provides important preliminary evidence on which recommendations for decisions about antibiotic treatment durations for exacerbations of bronchiectasis can be based. However, the study also has somemain limitations. First, there is a significant imbalance between patients treated with longer coursesas compared to shorter courses. Second, the microbiological study was performed during an exacerbation and we did not establish whether the identified microorganisms were responsible for prior chronic bronchial infection. Third, we could not perform a complete microbiological study in all cases, despite almost all patients providing at least one respiratory sample. Fourth, the present study was not powered to detect differences in mortality rates because this was not a study goal. Nevertheless, based on the 1-year follow-up results, we speculate that duration of antibiotic treatment does not affect the mortality rate.

## 5. Conclusions

In conclusion, LTOT use, the presence of a moderate to severe exacerbation, and microbiological isolation of *P. aeruginosa* each are associated with the need for a longer course of antibiotic therapy during bronchiectasis exacerbations. Patients receiving longer courses of treatment also tend to have more severe disease, colonization with *P. aeruginosa*, and a history of worse outcomes in terms of previous hospitalizations. Accordingly, decisions about the duration of antibiotic therapy should be guided by complete clinical and microbiological assessments of patients when they present with an acute exacerbation. Our study is really the reflection of clinical practice and if on the one hand it has a potential bias in the process of treatment decision-making, on the other it provides insights into a subset of exacerbated patients who could benefit from longer antibiotic treatment. However, more evidence is still needed to support clinic decision-making. Studies are currently underway to determine the optimal length of treatment in CF, and similar studies are needed for patients with non-CF bronchiectasis.

## Figures and Tables

**Table 1:** Baseline characteristics.

	All Patients	SC ≤ 14 Days	LC 15–21 Days	*p*-Value ^a^
**Patients**	*n* = 191	*n* = 132	*n* = 59	
**Clinical**				
Female sex, *n* (%)	108 (56.5)	76 (57.6)	32 (54.2)	0.667
Age, median (Q1; Q3), years	72 (63; 79)	72 (62.5; 79)	73 (64; 78)	0.881
BMI, median (Q1; Q3), Kg/m^2^	25.3 (22.2; 28.9)	25.5 (22.3; 29.4)	25 (21.9; 28.9)	0.576
Smoking habit, *n* (%)				0.738
Actual	7 (3.7)	5 (3.8)	2 (3.4)	
Former	83 (43.5)	55 (41.7)	28 (47.5)	
Influenza vaccination, *n* (%)	130 (68.4)	94 (71.8)	36 (61)	0.141
Pneumococcal vaccination, *n* (%)	93 (57.4)	67 (59.8)	26 (52)	0.352
Etiology, *n* (%)				0.535
Post-infectious	65 (34)	49 (37.1)	16 (27.1)	
Idiopathic	61 (32)	38 (28.8)	23 (39)	
Asthma	13 (6.8)	8 (6.1)	5 (8.5)	
COPD	36 (18.8)	25 (18.9)	11 (18.6)	
Others	16 (8.4)	12 (0.08)	4 (6.8)	
Comorbidities, *n* (%) ^b^				
Arterial hypertension	93 (48.7)	59 (44.7)	34 (57.6)	0.099
Arrhythmia	20 (17.2)	17 (22.7)	3 (7.3)	0.036
Dyslipidemia	43 (37.1)	31 (41.3)	12 (29.3)	0.198
Diabetes	22 (11.5)	14 (10.6)	8 (13.6)	0.555
Therapy, *n* (%) ^c^				
Mucolytics	76 (39.8)	47 (35.6)	29 (49.2)	0.077
Inhaled Antibiotic	43 (22.5)	27 (20.5)	16 (27.1)	0.308
Bronchodilators	263 (68.8)	179 (67.8)	84 (71.18)	0.268
Inhaled steroid	154 (80.6)	108 (81.8)	13 (22)	0.534
Oral corticosteroids (<28 days)	16 (8.4)	9 (6.8)	7 (11.9)	0.418
LTOT	26 (13.6)	11 (8.3)	15 (25.4)	0.001
**Pulmonary function**				
FVC pre-BD, median (Q1; Q3), %	82.5 (67; 99.3)	81.5 (69; 103)	84 (66; 96.3)	0.498
FEV_1_ pre-BD, median (Q1; Q3), %	61 (44.8; 76.6)	63 (45; 79)	58 (41; 68.6)	0.181
FEV_1_/FVC pre-BD, median (Q1; Q3), %	57.3 (49.8; 65)	58 (49; 65)	56.5 (50; 62.50)	0.546
**Severity**				
Lobes affected (HRCT), *n* (%)				0.312
<3	77 (41)	56 (43.4)	21 (35.6)	
≥3	111 (59)	73 (56.6)	38 (64.4)	
Type of BE (HRCT), *n* (%)				<0.001
Cylindrical	58 (30.7)	33 (25.4)	25 (42.4)	0.019
Varicose	12 (6.3)	7 (5.4)	5 (8.5)	0.521
Cystic	13 (6.9)	5 (3.8)	8 (13.6)	0.026
Chronic colonization, *n* (%)				
*Pseudomonas aeruginosa*	89 (46.6)	52 (39.4)	37 (62.7)	0.003
Others	46 (24.3)	31 (23.8)	15 (25.4)	0.815
History of exacerbations due to BE (1 year), *n* (%)	143 (75.3)	94 (71.8)	49 (83)	0.095
History of pneumonia (1 year), *n* (%)	32 (16.8)	25 (19.1)	7 (11.9)	0.218
History of hospitalizations due to BE (1 year), *n* (%)	97 (50.8)	60 (45.5)	37 (62.7)	0.028
BSI stage, *n* (%)				0.315
Mild 0–4	19 (9.9)	16 (12.1)	3 (5.1)	
Moderate 5–8	45 (23.6)	31 (23.5)	14 (23.7)	
Severe ≥9	127 (66.5)	85 (64.4)	42 (71.2)	
FACED score, *n* (%)				0.029
Mild 0–2	94 (49.2)	72 (54.5)	22 (37.3)	0.027
Moderate 3–4	61 (31.9)	41 (31.1)	20 (33.9)	0.697
Severe 5–7	36 (18.8)	19 (14.4)	17 (28.8)	0.019

**Abbreviations:** BD, bronchodilator; BE, bronchiectasis; BMI, body mass index; BSI, Bronchiectasis Severity Index; COPD, chronic obstructive pulmonary disease; FEV_1_, Forced expiratory volume in 1 second; FVC, Forced Volume Vital Capacity; HRCT, high-resolution computed tomography; LC, long-course; LTOT, Long-Term Oxygen Therapy; SC, short-course. Percentages calculated on non-missing data. ^a^ The *p*-values correspond to differences between the two groups (short-course group (≤14 days) vs. long-course group (15–21 days)). ^b^ Could have more than one medication. ^c^ Could have more than one comorbidity.

**Table 2:** Characteristics during exacerbation.

	All Patients	SC ≤ 14 Days	LC 15–21 Days	*p*-Value ^a^
**Patients**	*n* = 191	*n* = 132	*n* = 59	
**Clinical variables, *n* (%)**				
Increased sputum volume	156 (83.4)	106 (82.8)	50 (84.7)	0.741
Increased sputum purulence	135 (72.2)	91 (71.1)	44 (74.6)	0.621
Increased dyspnea	145 (77.5)	98 (76.6)	47 (79.7)	0.637
Fever (>38 °C)	109 (58.3)	79 (61.7)	30 (50.8)	0.161
Chest pain	45 (24.1)	39 (30.5)	6 (10.2)	0.003
Hemoptysis	15 (8)	9 (7)	6 (10.2)	0.563
Increased cough	159 (85)	108 (84.4)	51 (86.4)	0.713
**Blood analysis, median (Q1; Q3)**				
C-Reactive Protein, mg/dL	5.8 (1.9; 15.5)	5.6 (2; 16.3)	6.5 (1.5; 13.1)	0.988
Hemoglobin, g/L	106 (13; 129)	113 (13.7; 133)	114.1 (12.3; 115)	0.232
Platelets, 10^9^/L	240 (202; 310)	245 (204; 303)	236 (200.5; 325.5)	0.674
Leukocytes, 10^9^/L	10.7 (8.1; 14.3)	10.6 (8.4; 13.8)	11.4 (7.5; 14.9)	0.810
PaO_2_	64.7 (57; 73.4)	63 (56.5; 72.7)	67.3 (58.4; 73.4)	0.327
**Severity**				
Severity of exacerbation				0.005
Mild	48 (25.1)	42 (31.8)	6 (10.2)	0.001
Moderate to severe	143(74.9)	90 (61.2)	53 (89.8)	0.001
Site of treatment				0.013
Outpatient treatment	52 (27.2)	43 (32.6)	9 (15.3)	
Hospital ward/Intensive Care Unit/Intermediate Care Unit	139 (72.8)	89 (67.4)	50 (84.7)	
Duration of exacerbation, median (Q1; Q3), days	11 (8; 15)	10 (7; 14)	15 (11; 20)	<0.001
Duration of hospitalization, median (Q1; Q3), days	8 (6; 11)	8 (6; 10)	9.5 (7;17)	0.002
Diagnosis of CAP, *n* (%)	49 (25.7)	31 (23.5)	18 (30.5)	0.304
Complications, *n* (%)^b^				
Intubation/MV	4 (2.1)	2 (1.5)	2 (3.4)	0.589
NIV	7 (3.7)	3 (2.3)	4 (6.8)	0.205
Sepsis	7 (3.7)	3 (2.3)	4 (6.8)	0.205
Septic shock	4 (2.1)	1 (0.8)	3 (5.1)	0.088
Acute myocardial infarction	2 (1)	0	2 (3.4)	0.094
Arrhythmia	8 (4.2)	6 (4.5)	2 (3.4)	>0.999
**Microbiology, *n* (%)**				
Patients with defined etiology	141 (73.8)	90 (68.2)	51 (86.4)	0.008
*Haemophilus influenzae* ^c^	12 (8.5)	11 (12.2)	1 (2)	0.056
*Escherichia coli* ^c^	5 (3.5)	4 (4.4)	1 (2)	0.654
*Klebsiella pneumoniae* ^c^	1 (0.7)	1 (1.1)	0	>0.999
*Moraxella catarrhalis* ^c^	3 (2.1)	3 (3.3)	0	0.553
*Pseudomonas aeruginosa* ^c^	28 (19.9)	12 (13.39)	16 (31.4)	0.010
*Staphylococcus aureus* ^c^	2 (1.4)	0	2 (3.9)	0.129
*Mycoplasma pneumoniae* ^c^	2 (1.4)	2 (2.2)	0	0.535
*Streptococcus pneumoniae* ^c^	11 (7.8)	6 (6.7)	5 (9.8)	0.527
Virus ^c^	6 (4.3)	5 (5.69)	1 (2)	0.418
Other ^c^	17 (12.1)	13 (14.4)	4 (7.8)	0.247
Polymicrobial ^c^	52 (36.9)	32 (35.69)	20 (39.2)	0.665
Antibiotic treatment				
Number of initial antibiotics, *n* (%)				<0.001
1	94 (49.2)	75 (56.8)	19 (32.2)	0.002
2	74 (38.7)	49 (37.1)	25 (42.4)	0.491
≥3	23 (12)	8 (6.1)	15 (25.4)	<0.001

**Abbreviations:** CAP, Community-acquired pneumonia; i.v., intravenous; LC, long-course; MV, Mechanical ventilation; NIV, Noninvasive ventilation; PaO_2_, partial pressure of oxygen; SC, short-course. Percentages calculated on non-missing data. ^a^ The *p*-values correspond to differences between the two groups (short-course group (≤14 days) vs. long-course group (15–21 days)). ^b^ Could have more than one complication. ^c^ The percentages of pathogens are related to the number of patients with etiologic diagnosis in each group.

**Table 3:** Outcomes at 1 year.

	All Patients	SC ≤14 Days	LC 15–21 Days	*p*-Value ^a^
**Patients**	*n* = 191	*n* = 132	*n* = 59	
**Outcomes**				
Poor clinical response, *n* (%)	36 (22.2)	18 (15.9)	18 (36.7)	0.003
History of exacerbations for 1 year, *n* (%)	114 (61.3)	80 (62.5)	34 (58.6)	0.615
History of hospitalization for 1 year, *n* (%)	49 (50.5)	29 (48.3)	20 (54.1)	0.584
Mortality during the exacerbation, *n* (%)	3 (1.6)	1 (0.8)	2 (3.4)	0.176
Mortality after 30 days, *n* (%)	1 (0.5)	1 (0.8)	0	0.501
Mortality after 1 year, *n* (%)	16 (8.4)	12 (9.2)	4 (6.8)	0.779

**Abbreviations:** LC, long-course; SC, short-course. ^a^ The *p*-values correspond to differences between the two groups (short-course group (≤14 days) vs. long-course group (15–21 days)).

**Table 4:** Univariate and multivariable logistic regression analyses in predicting long-course antibiotic treatment.

Variable	Univariate			Multivariable ^a,b,c^		
	OR	95% CI	*p*-Value	OR	95% CI	*p*-Value
LTOT	3.75	1.60–8.78	0.002	2.51	1.02–6.22	0.046
FACED score			0.033	–	–	–
Mild 0–2	1.00	–	–	–	–	–
Moderate 3–4	1.60	0.78–3.27	0.20	–	–	–
Severe 5–7	2.93	1.30–6.58	0.009	–	–	–
Site of treatment: Hospital ward/Intensive Care Unit/Intermediate Care Unit	2.68	1.21–5.96	0.015	–	–	–
Moderate to severe exacerbation	4.12	1.64–10.35	0.003	3.31	1.26–8.65	0.015
*Pseudomonas aeruginosa*	3.76	1.94–7.29	<0.001	2.89	1.43–5.80	0.003
*MRSA*	6.02	1.13–31.98	0.035	–	–	–

**Abbreviations:** CI, confidence interval; i.v., intravenous; LC, long-course; LTOT, Long-Term Oxygen Therapy; MRSA, Methicillin-resistant *Staphylococcus aureus*; MV, mechanical ventilation; OR, odds ratio. Data are shown as estimated ORs (95% CIs) of the explanatory variables in the LC group. The OR represents the odds that LC antibiotic treatment will occur, given exposure to the explanatory variable, compared to the odds of the outcome occurring in the absence of that exposure. *p*-values are based on the null hypothesis that all ORs relating to an explanatory variable equal unity (i.e., no effect). ^a^ Adjusted for center. ^b^ Hosmer–Lemeshow goodness-of-fit test, *p* = 0.35 ^c^ Probability of LC antibiotic treatment = Exp (β)/(1 + Exp (β)), where β = −2.543 + 0.528 (for Valencia center) + 0.922 (for LTOT) + 1.196 (for moderate to severe exacerbation) + 1.061 (for *P. aeruginosa* microbiology). In the presence of all these risks factors, the probability of LC antibiotic treatment was 76.2%, while in the absence of all these risk factors, the probability of LC antibiotic treatment was 7.3%.

## Supplementary Materials

The Supplementary Materials are available online at https://www.mdpi.com/2077-0383/8/11/1950/s1.

## Abbreviations

AUC	Area under the curve
BSI	Bronchiectasis Severity Index
CF	Cystic fibrosis
CI	Confidence interval
COPD	Chronic obstructive pulmonary disease
CT	Computed tomography
LC	Long-course (15–21 days)
LTOT	Long-term oxygen therapy
OR	Odds ratio
SC	Short-course (≤14 days)

## References

[1] Watt A.P., Brown V., Courtney J., Kelly M., Garske L., Elborn J.S., Ennis M. (2004). Neutrophil apoptosis, proinflammatory mediators and cell counts in bronchiectasis. Thorax.

[2] Bell S.C., Elborn J.S., Byrnes C.A. (2018). Bronchiectasis: Treatment decisions for pulmonary exacerbations and their prevention. Respirology.

[3] Chalmers J.D., Goeminne P., Aliberti S., McDonnell M.J., Lonni S., Davidson J., Poppelwell L., Salih W., Pesci A., Dupont L.J. (2014). The bronchiectasis severity index. An international derivation and validation study. Am. J. Respir. Crit. Care Med..

[4] Hill A.T., Haworth C.S., Aliberti S., Barker A., Blasi F., Boersma W., Chalmers J.D., De Soyza A., Dimakou K., Elborn J.S. (2017). Pulmonary exacerbation in adults with bronchiectasis: A consensus definition for clinical research. Eur. Respir. J..

[5] Martínez-García M.Á., Máiz L., Olveira C., Girón R.M., de la Rosa D., Blanco M., Cantón R., Vendrell M., Polverino E., de Gracia J. (2018). Normativa sobre la valoración y eldiagnóstico de lasbronquiectasias en el adulto. Arch. Bronconeumol..

[6] Polverino E., Goeminne P.C., McDonnell M.J., Aliberti S., Marshall S.E., Loebinger M.R., Murris M., Cantón R., Torres A., Dimakou K. (2017). European Respiratory Society guidelines for the management of adult bronchiectasis. Eur. Respir. J..

[7] Bilton D., Henig N., Morrissey B., Gotfried M. (2006). Addition of Inhaled Tobramycin to Ciprofloxacin for Acute Exacerbations of Pseudomonas aeruginosa Infection in Adult Bronchiectasis. Chest.

[8] Martínez-García M.Á., Máiz L., Olveira C., Girón R.M., de la Rosa D., Blanco M., Cantón R., Vendrell M., Polverino E., de Gracia J. (2018). Spanish Guidelines on the Evaluation and Diagnosis/Treatment of Bronchiectasis in Adults. Arch. Bronconeumol..

[9] Cantón R., Máiz L., Escribano A., Olveira C., Oliver A., Asensio O., Gartner S., Roma E., Quintana-Gallego E., Salcedo A. (2015). Spanish Consensus on the Prevention and Treatment of *Pseudomonas aeruginosa* Bronchial Infections in Cystic Fibrosis Patients. Arch. Bronconeumol..

[10] Wilson R., Anzueto A., Miravitlles M., Arvis P., Faragó G., Haverstock D., Trajanovic M., Sethi S. (2011). A novel study design for antibiotic trials in acute exacerbations of COPD: MAESTRAL methodology. Int. J. Chron. Obstruct. Pulmon. Dis..

[11] Martínez-García M.Á., de Gracia J., Vendrell Relat M., Girón R.M., Máiz Carro L., de la Rosa Carrillo D., Olveira C. (2014). Multidimensional approach to non-cystic fibrosis bronchiectasis: The FACED score. Eur. Respir. J..

[12] Hosmer D.W., Lemeshow S. (2000). Applied Logistic Regression.

[13] Sterne J.A., White I.R., Carlin J.B., Spratt M., Royston P., Kenward M.G., Wood A.M., Carpenter J.R. (2009). Multiple imputation for missing data in epidemiological and clinical research: Potential and pitfalls. Br. Med. J..

[14] Efron B., Tibshirani R. (1994). An Introduction to the Bootstrap.

[15] Chalmers J.D., Aliberti S., Filonenko A., Shteinberg M., Goeminne P.C., Hill A.T., Fardon T.C., Obradovic D., Gerlinger C., Sotgiu G. (2018). Characterization of the “Frequent Exacerbator Phenotype” in Bronchiectasis. Am. J. Respir. Crit. Care Med..

[16] Bhatt J.M. (2013). Treatment of pulmonary exacerbations in cystic fibrosis. Eur. Respir. Rev..

[17] Murray M.P., Turnbull K., MacQuarrie S., Hill A.T. (2008). Assessing response to treatment of exacerbations of bronchiectasis in adults. Eur. Respir. J..

[18] Chalmers J.D., Smith M.P., McHugh B.J., Doherty C., Govan J.R., Hill A.T. (2012). Short- and long-term antibiotic treatment reduces airway and systemic inflammation in non-cystic fibrosis bronchiectasis. Am. J. Respir. Crit. Care Med..

[19] Collaco J.M., Green D.M., Cutting G.R., Naughton K.M., Mogayzel P.J. (2010). Location and Duration of Treatment of Cystic Fibrosis Respiratory Exacerbations Do Not Affect Outcomes. Am. J. Respir. Crit. Care Med..

[20] Hill A.T., Sullivan A.L., Chalmers J.D., De Soyza A., Elborn S.J., Floto A.R., Grillo L., Gruffydd-Jones K., Harvey A., Haworth C.S. (2019). British Thoracic Society Guideline for bronchiectasis in adults. Thorax.

[21] Rosales-Mayor E., Polverino E., Raguer L., Alcaraz V., Gabarrus A., Ranzani O., Menendez R., Torres A. (2017). Comparison of two prognostic scores (BSI and FACED) in a Spanish cohort of adult patients with bronchiectasis and improvement of the FACED predictive capacity for exacerbations. PLoS ONE.

[22] Crisafulli E., Ielpo A., Barbeta E., Ceccato A., Huerta A., Gabarrús A., Soler N., Chetta A., Torres A. (2018). Clinical variables predicting the risk of a hospital stay for longer than 7 days in patients with severe acute exacerbations of chronic obstructive pulmonary disease: A prospective study. Respir. Res..

[23] Sundh J., Ahmadi Z., Ekström M. (2018). Daily duration of long-term oxygen therapy and risk of hospitalization in oxygen-dependent COPD patients. Int. J. Chron. Obstruct. Pulmon. Dis..

[24] Martínez-García M.A., Soler-Cataluña J.J., Perpiñá-Tordera M., Román-Sánchez P., Soriano J. (2007). Factors associated with lung function decline in adult patients with stable non-cystic fibrosis bronchiectasis. Chest.

[25] Wilson C.B., Jones P.W., O’Leary C.J., Hansell D.M., Cole P.J., Wilson R. (1997). Effect of sputum bacteriology on the quality of life of patients with bronchiectasis. Eur. Respir. J..

