# Supplementary material for: Clinical Factors Associated with a Shorter or Longer Course of Antibiotic Treatment in Patients with Exacerbations of Bronchiectasis: A Prospective Cohort Study
Source: J Clin Med. 2019 Nov 12;8(11):1950. doi: 10.3390/jcm8111950 (PMC6912316; doi:10.3390/jcm8111950)
Supplement: Supplementary file 1 [file jcm-08-01950-s001.zip › Supplementary material.docx]

**SUPPLEMENTARY INFORMATION**

**Clinical factors associated with a shorter or longer course of antibiotic treatment in patients with exacerbations of bronchiectasis: a prospective cohort study.**

*Giulia Scioscia^1,2^, *Rosanel Amaro^2,3^, Victoria Alcaraz^3^, Albert Gabarrús^3^, Patricia Oscanoa^2^, Laia Fernandez^3^, Rosario Menendez^4^, Raul Mendez^4^, Maria Pia Foschino Barbaro^1^, Antoni Torres^2, 3^.

^1^Medical and Surgical Sciences Department, Institute of Respiratory Disease, University of Foggia, Foggia (Italy).

^2^Institut ClínicRespiratori, Hospital Clínic de Barcelona (Spain).

^3^Fundació Clínic per la RecercaBiomèdica (FCRB), IDIBAPS, CIBERES. Hospital Clínic de Barcelona (Spain).

^4^Pneumology Department, La Fe University and Polytechnic Hospital. La Fe Health Research Institute. Valencia (Spain).

*co-first authors

**Appendix S1-** Factors associated with longer courses of antibiotic treatment after excluding bronchiectasis exacerbated patients with a diagnosis of community-acquired pneumonia.

**Figure S1***-* Area under the receiver-operator characteristic curve for the multivariable model (cohort without CAP)

*
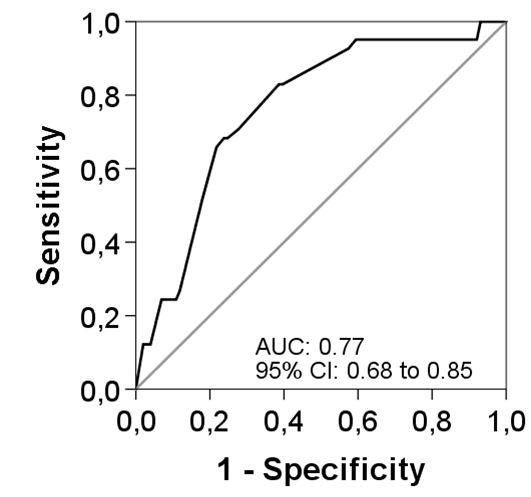
*

**Table S1.** Univariate and multivariable logistic regression analyses in predicting long-course antibiotic treatment (cohort without CAP).

| **Variable** | **Univariate** | | | **Multivariable^a, b, c^** | | |
| --- | --- | --- | --- | --- | --- | --- |
|  | **OR** | **95% CI** | **P** | **OR** | **95% CI** | **P** |
| **LTOT** | 2.94 | 1.12-7.72 | 0.029 | - | - | - |
| **FACED score** |  |  | 0.030 |  |  | 0.014 |
| Mild 0–2 |  |  | - |  |  | - |
| Moderate 3–4 | 1.49 | 0.62-3.60 | 0.37 | 2.97 | 1.09-8.14 | 0.034 |
| Severe 5–7 | 3.48 | 1.38-8.79 | 0.008 | 4.31 | 1.49-12.42 | 0.007 |
| **Arrhythmia** | 0.28 | 0.08-1.01 | 0.051 | 0.19 | 0.05-0.73 | 0.016 |
| **Fever** | 0.49 | 0.24-1.04 | 0.062 | - | - | - |
| **Site of treatment: Hospital ward / Intensive Care Unit / Intermediate Care Unit** | 3.06 | 1.23-7.57 | 0.016 | - | - | - |
| **Moderate to severe exacerbation** | 3.52 | 1.35-9.14 | 0.010 | - | - | - |
| ***Pseudomonas aeruginosa*** | 3.17 | 1.49-6.76 | 0.003 | 3.07 | 1.31-7.18 | 0.010 |
| ***MRSA*** | 5.35 | 0.94-30.45 | 0.059 | - | - | - |

**Abbreviations.**CI, confidence interval; LC, long-course; LTOT, Long-Term Oxygen Therapy; MRSA, Methicillin-resistant *Staphylococcus aureus*; OR, odds ratio.

Data are shown as estimated ORs (95% CIs) of the explanatory variables in the LC group. The OR represents the odds that LC antibiotic treatment will occur, given exposure to the explanatory variable, compared to the odds of the outcome occurring in the absence of that exposure. P-values are based on the null hypothesis that all ORs relating to an explanatory variable equal unity (i.e., no effect).

^a^ Adjusted for center.

^b^ Hosmer–Lemeshow goodness-of-fit test, p = 0.074

^c^ Probability of LC antibiotic treatment = Exp (β) / (1 + Exp (β)), where β = -2.256 + 0.874 (for Valencia center) + 1.089 (for moderate FACED score) + 1.460 (for severe FACED) – 1.647 (for arrhythmia) + 1.120 (for *P.aeruginosa* microbiology).

**Table S2.** Outcomes (cohort without CAP)

|  | **All patients** | **SC**  **≤14 days** | **LC**  **15–21 days** | **P value** |
| --- | --- | --- | --- | --- |
| **Patients** | N=142 | N=101 | N=41 |  |
| **Outcomes** |  |  |  |  |
| Poor clinical response, n (%) | 19 (23.2) | 8 (15.4) | 11 (36.7) | 0.003 |
| History of exacerbations for 1 year, n (%) | 84 (60.9) | 61 (62.2) | 23 (57.5) | 0.604 |
| History of hospitalization for 1 year, n (%) | 40 (52.6) | 23 (47.9) | 17 (60.7) | 0.281 |
| Mortality during the exacerbation, n (%) | 3 (3.1) | 1 (1.6) | 2 (5.9) | 0.250 |
| Mortality after 30 days, n (%) | 1 (1) | 1 (1.6) | 0 | 0.457 |
| Mortality after 1 year, n (%) | 9 (9.4) | 6 (9.7) | 3 (8.8) | 0.891 |

**Abbreviations.**LC, long-course; SC, short-course.
